# Risk indices predicting graft use, graft and patient survival in solid pancreas transplantation: a systematic review

**DOI:** 10.1186/s12876-021-01655-2

**Published:** 2021-02-23

**Authors:** Jonathan E. H. Ling, Timothy Coughlan, Kevan R. Polkinghorne, John Kanellis

**Affiliations:** 1 Department of Nephrology, Monash Medical Centre, Monash Health, 246 Clayton Road, Clayton, Melbourne, VIC 3168 Australia; 2 Centre for Inflammatory Diseases, Department of Medicine, Monash University, Clayton, Melbourne, Australia; 3 Department of Renal Services, Latrobe Regional Hospital, Victoria, Australia; 4 Department of Epidemiology and Preventive Medicine, School of Public Health and Preventive Medicine, Monash University, Prahran, Melbourne, Australia

**Keywords:** Risk index, Pancreas, Transplantation, Survival, Organ acceptance, Risk

## Abstract

**Background:**

Risk indices such as the pancreas donor risk index (PDRI) and pre-procurement pancreas allocation suitability score (P-PASS) are utilised in solid pancreas transplantation however no review has compared all derived and validated indices in this field. We systematically reviewed all risk indices in solid pancreas transplantation to compare their predictive ability for transplant outcomes.

**Methods:**

Medline Plus, Embase and the Cochrane Library were searched for studies deriving and externally validating risk indices in solid pancreas transplantation for the outcomes of pancreas and patient survival and donor pancreas acceptance for transplantation. Results were analysed descriptively due to limited reporting of discrimination and calibration metrics required to assess model performance.

**Results:**

From 25 included studies, discrimination and calibration metrics were only reported in 88% and 38% of derivation studies (n = 8) and in 25% and 25% of external validation studies (n = 12) respectively. 21 risk indices were derived with mild to moderate ability to predict risk (C-statistics 0.52–0.78). Donor age, donor body mass index (BMI) and donor gender were the commonest covariates within derived risk indices. Only PDRI and P-PASS were subsequently externally validated, with variable association with post-transplant outcomes. P-PASS was not associated with pancreas graft survival.

**Conclusion:**

Most of the risk indices derived for use in solid pancreas transplantation were not externally validated (90%). PDRI and P-PASS are the only risk indices externally validated for solid pancreas transplantation, and when validated without reclassification measures, are associated with 1-year pancreas graft survival and donor pancreas acceptance respectively. Future risk indices incorporating recipient and other covariates alongside donor risk factors may have improved predictive ability for solid pancreas transplant outcomes.

## Background

Risk indices are used to predict risk and guide decision-making during various types of organ transplantation [1–5]. Comprising a combination of donor, recipient and transplant-related factors, risk indices are used to prioritise patients on the transplant waiting list for transplantation [1] as well as to guide donor organ acceptance for transplantation [4, 6]. Risk indices currently in use for solid pancreas transplantation are the pre-procurement pancreas allocation suitability score (P-PASS) and the pancreas donor risk index (PDRI), both comprised primarily of donor factors [3–5]. The P-PASS is currently used by countries within the Eurotransplant network to guide donor pancreas acceptance [6] while PDRI predicts pancreas graft survival and is reported in pancreas transplantation within the USA [7]. However, they are not widely used as external validation studies have reported varying association with their intended outcomes [8–11]. Other risk indices have been derived for use in pancreas transplantation however have not been validated widely in external cohorts [12, 13].

We compared the predictive ability of all current risk indices derived for use in solid pancreas transplantation via a systematic review. This would guide future work in incorporating a risk index into the Australian and New Zealand pancreas transplant protocol, as no index is currently used to guide solid pancreas transplantation locally [5, 14].

## Methods

This systematic review was guided by the Cochrane’s Critical Appraisal and Data Extraction for Systematic Reviews of Prediction Modelling Studies (CHARMS) tool for reviews of prediction modelling studies [15] and used the Transparent Reporting of a multivariable prediction model for Individual Prognosis or Diagnosis (TRIPOD) checklist to assess the completeness of individual study reporting [16]. This project was exempt from requiring local ethics board approval as only previously published data (no identifiable individual data) was the subject of review. The review protocol was registered via the International Prospective Register of Systematic Reviews (PROSPERO ID CRD42018080189) [17].

### Literature search

Ovid Medline, Embase and the Cochrane Database of Systematic Reviews were searched for studies which derived or validated risk indices used in pancreas transplantation from inception to the 30^th^ of March 2020. Grey literature searching of OpenGrey, Scopus and Web of Science was performed for the same period. Search terms included the following keywords and MESH terms; pancreas, transplant, donor, recipient, index or indices, model or models, tool or tools, pancreas after kidney (PAK), pancreas transplant alone (PTA), simultaneous kidney-pancreas transplant (SPK), P-PASS and PDRI. (Search protocols in Additional file 1: Supplement 1).

### Eligibility criteria

All observational studies which derived or validated risk indices for solid pancreas transplantation were accepted for full-text review. Islet transplant studies were excluded. A risk index or model was defined as a combination of multiple predictors which calculated individual patient risk of a future outcome [18]. Studies examining a single risk factor’s association with solid pancreas transplant outcomes were excluded. Likewise, case series and studies identifying factors associated with solid pancreas transplant outcomes without deriving a risk index or validating a known risk index were excluded. Studies whose aims did not include either deriving or validating a risk index but analysed PDRI or P-PASS for association with various pancreas transplant outcomes were retained for discussion but not included in the analysis. We anticipated a limited number of relevant studies and therefore included abstracts meeting inclusion criteria where no full-text article was available.

### Study outcomes

Primary outcomes were pancreas graft survival, patient survival and donor pancreas acceptance for solid-organ pancreas transplantation. Pancreatic graft failure was defined as a permanent return to insulin therapy or pancreatectomy [14] and was reported as death-censored where possible based on study reporting.

### Data extraction and critical appraisal

The CHARMS tool was utilized for data extraction (as data was non-randomised) [15]. Domains within CHARMS include data source, participant description, predicted outcomes, significant predictors, sample size, data handling, model development, model performance, model evaluation and results. The TRIPOD checklist was used to assess the quality of data reporting for all included studies [16]. The Prediction Model Risk of Bias Assessment Tool (PROBAST) was used to assess risk of bias and applicability of included studies [19, 20].

Model performance was assessed via study reporting of discrimination and calibration metrics [21], as described in TRIPOD, PROBAST and elsewhere [16, 20, 22, 23]. Discrimination (measured via the C-statistic or the area under the receiver operating characteristic (AUROC) curve [22, 23]) is the ability to distinguish those at higher risk (for outcome of choice) from those at lower risk. For instance, a C-statistic of 1.0 indicates an index is able to perfectly predict subjects at higher (or lower) risk, whereas 0.5 represents inability to differentiate between risk outcomes (akin to flipping a coin) [23]. The accuracy of an indices’ predicted risk (compared to the actual absolute risk) is measured by calibration [22, 23]. This is performed via the Hosmer–Lemeshow test, comparison of calibration plots or observed-predicted outcome ratios [22–24]. A well-calibrated model is denoted by the lack of significant differences between observed and predicted outcomes or a Hosmer–Lemeshow p value of > 0.05 [24, 25].

Model predictors, effect estimates (hazard ratios), missing data, events-per-variable (EPV) rate were extracted to assess study quality [15, 26]. Risk indices derived in a cohort with an EPV < 10 have a risk of overfitting (small number of outcome events compared to number of model predictors) [22, 23]. When indices were externally validated, we noted if reclassification of predictors took place [27]. If a single study derived more than one risk index, they were considered unique models if the predictors within the models were different.

Two authors (JEHL and TC) performed title, abstract and full-text reviews independently and compared results. Where there was no consensus between both authors, a third author (JK or KRP) was involved. Data extraction and risk of bias assessment was performed by two authors (JEHL and TC) independently and results were compared. Study authors were contacted for clarification and data extraction (9 study authors contacted) particularly when only abstract-level data was available, but only one response was forthcoming.

### Data analysis

All risk indices derived for use in solid pancreas transplantation were grouped by the outcomes they were derived to predict. If more than two studies derived risk indices for similar outcomes, we intended to meta-analyse their metrics of model performance. Unfortunately, insufficient metrics of discrimination and calibration were reported by studies deriving and externally validating risk indices to allow pooling of these metrics in a meta-analysis.

Results are therefore presented in two analyses. Firstly, we describe all risk indices derived to predict our primary outcomes in solid pancreas transplantation. Secondly, we describe the external validation of these risk indices. For each analysis, we report risk index performance via their discrimination and calibration metrics (where present) and assess their method of derivation, association with outcome, study quality and risk of bias. We used the Grading of Recommendations, Assessment, Development and Evaluations (GRADE) framework to summarize the current evidence for the use of externally validated risk indices by outcome, according to the domains of Risk of bias, Inconsistency, Indirectness, Imprecision and Publication bias [28–30].

## Results

**Fig. 1:**
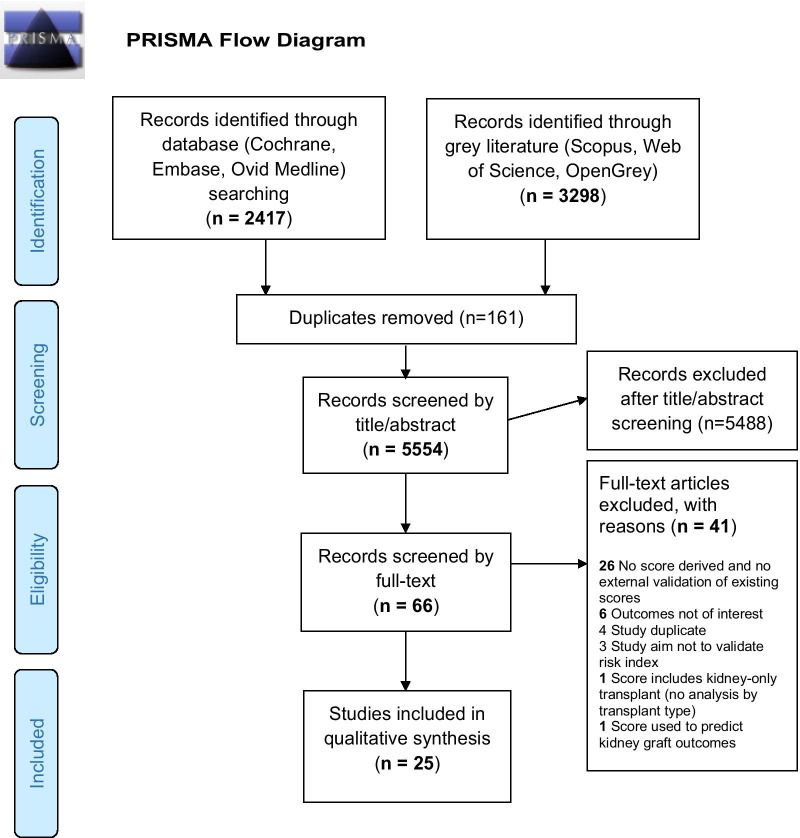
PRISMA flow diagram

5715 abstracts were identified. After deduplication, 5554 studies underwent title/abstract screening and 66 studies proceeded to full-text screening. After further exclusions, 25 studies were included in the review. The PRISMA (Preferred Reporting Items for Systematic Reviews and Meta-Analyses) flow diagram [31] is included in Fig. 1. Eight studies derived 21 risk indices predicting our primary outcomes (including PDRI and P-PASS) [3, 4, 11–13, 32–35] (Table 1). From these derived risk indices, only PDRI and P-PASS (Table 2) were externally validated in 19 studies. Regarding TRIPOD assessment of completeness of data recording for all studies, only 20 of all 33 domains recorded > 70% adherence (Additional file 1: Supplement 2).

**Table 1:** Studies deriving risk indices for solid organ pancreas transplantation

Study, year (transplant type)	Risk index (intended use)	Cohort size, source (study dates)	Outcome	Variables entered	Final model variables	Model type	Discrimination	Calibration*
Axelrod D 2010 (All) [3]	Pancreas donor risk index (pre-transplant to inform donor organ acceptance)	9401 pancreas recipients from SRTR registry, USA (2000–2006)	1-year pancreas graft survival (All)	**Donor**: age, BMI, gender, race, height, cause of death, DCD and creatinine **Recipient**: age, BMI, gender, race, PRA, previous PVD, primary payment type, albumin, previous transplant **Other**: transplant centre, duct management, degree of HLA matching, preservation time and transplant year	Donor age, BMI, gender, black or Asian race, cause of death, creatinine, height, DCD status, CIT, PAK transplant with a CVA donor	Cox regression model (n = 1)	0.67	Observed: 1351 Predicted: NI
Dorsey SG 1997 (All) [13]	Logistic regression model and a neural network model (pre-transplant)	160 pancreas recipients from single centre, USA (1991–1996)	3-month pancreas survival (All)	**Donor**: age **Recipient**: age, blood transfusions, smoking and alcohol history, diabetes duration, RRT pre-transplant **Other**: sex or weight mismatch, having a PAK/PTA transplant, use of nonlocal organ procurement centre, HLA-DR mismatch	All variables used	Logistic regression model (n = 1)	0.78 Model sensitivity 35.7%	Observed: 23 Predicted: NI H–L p = 0.74 R^2^ = 0.24
						Neural network model (n = 1)	Correctly predicted 92.5% of cases Model sensitivity 68% Model specificity 96%	Observed: 23 Predicted: NI R^2^ = 0.71
Finger EB 2013 (All) [12]	Composite risk model (pre-transplant)	1115 pancreas recipients from single centre, USA (1998–2011)	3-month death-censored pancreas failure (All)	**Donor**: age, gender, race, cause of death, drug/alcohol abuse, pancreatitis history, BMI, DCD status, terminal creatinine, amylase, time to death from admission, CIT, PDRI **Recipient**: age, gender, BMI, re-transplantation, previous vascular disease, pre-transplant dialysis, smoking status **Other**: PRA, number of HLA mismatches, type of exocrine drainage	Donor age, BMI, CIT, terminal creatinine, presence of bladder drainage	Cox regression model (n = 1)	0.6 (≥ 1 risk factors) 0.59 (≥ 2 risk factors) 0.52 (≥ 3 risk factors)	Observed: 10.2% graft failure Predicted: 12.8% (≥ 1 risk factor) 26.7% (≥ 2 risk factors) 42.9% (≥ 3 risk factors)
Grochowiecki T 2014 (SPK) [32]	Logistic regression model	112 pancreas recipients from single centre, Poland (1988–2010)	Patient survival (timepoint unclear) (SPK)	**Donor**: age, gender, cause of death **Recipient**: age, gender, diabetes duration, RRT type and duration **Other**: type and time of pancreas/kidney anastomosis, type of enteric anastomosis, total ischaemia time, immunosuppression type	Donor age, duration of pancreas anastomosis (vascular), dialysis duration	Logistic regression model (n = 1)	0.8	Observed: 14 Predicted: NI
Kasiske BL 2013 (All) [33]	12 Cox regression models (to model survival outcomes by transplant type for viability of prospective pooling of transplant types for future survival analysis)	6078 pancreas recipients from SRTR registry, USA (2003–2010)	1-year SPK graft survival	**Donor**: age, BMI, deceased donor, PVD at listing, cause of death **Recipient**: age, BMI, gender, race, duration of dialysis, age of diabetes diagnosis, whether working or hospitalized at time of transplant, previous PVD, PRA, HLA mismatch **Other**: CIT, retransplantation, if pre-emptive kidney transplant	**Donor**: age, BMI, gender, cause of death, PDRI **Recipient**: age, BMI, gender, race, preservation method, whether working or hospitalized at time of transplant, pre-emptive kidney transplant, diabetes duration, diabetes type, peripheral vascular disease history, terminal eGFR and eGFR on discharge, PRA, previous pancreas transplant **Other**: CIT	Cox regression models (n = 12)	0.63 (0.60–0.67)	H–L p = 0.44
			1-year PAK graft survival				0.63 (0.58–0.69)	H–L p = 0.32
			1-year PTA graft survival				0.68 (0.61–0.75)	H–L p = 0.6
			1-year SPK patient survival				0.62 (0.57–0.69)	H–L p = 0.77
			1-year PAK patient survival				0.75 (0.62–0.88)	H–L p = 0.83
			1-year PTA patient survival				0.78 (0.58–0.98)	H–L p = 0.74
			3-year SPK graft survival				0.59 (0.56–0.62)	H–L p = 0.38
			3-year PAK graft survival				0.6 (0.56–0.64)	H–L p = 0.81
			3-year PTA graft survival				0.66 (0.61–0.71)	H–L p = 0.68
			3-year SPK patient survival				0.64 (0.6–0.68)	H–L p = 0.92
			3-year PAK patient survival				0.68 (0.59–0.77)	H–L p = 0.71
			3-year PTA patient survival				0.76 (0.66–0.86)	H–L p = 0.24
Smigielska K 2018 (All) [11]	Logistic regression model (pre-transplant)	408 pancreas recipients, multicentre, Poland (1998–2015)	1-year pancreas graft survival (All)	No clear specification of all baseline variables (only donor variables considered)	Donor age, BMI	Logistic regression model (n = 1)	0.61 (0.56–0.66)	Observed: 268 Predicted: NI
Sousa M 2014 (SPK) [35]	Two Cox regression models (pre-transplant)	292 pancreas recipients, single centre, Brazil (2000–2010)	3-month pancreas survival	**Donor**: age, BMI, gender, creatinine, sodium, amylase, norepinephrine, cause of death **Recipient**: age, BMI, duration of dialysis, duration of diabetes, need for dialysis, gender, cyclosporine, use of induction therapy, type of preservation fluid **Other**: CIT of pancreas and kidney, sequence of transplantation (pancreas or kidney first), type of duodenal anastomosis and venous drainage	Donor age, recipient BMI, use of induction therapy, iliac venous drainage, pancreas implantation first	Cox regression model (n = 2)	0.72 (0.65–0.78)	Observed: 56 Predicted: NI
			1-year patient survival	**Donor**: age, BMI, gender, creatinine, sodium, amylase, norepinephrine, cause of death **Recipient**: age, BMI, duration of dialysis, duration of diabetes, need for dialysis, gender, cyclosporine, use of induction therapy, type of preservation fluid **Other**: CIT of pancreas and kidney, sequence of transplantation (pancreas or kidney first), type of duodenal anastomosis and venous drainage	Recipient BMI, use of induction therapy	Cox regression model (n = 2)	0.67 (0.59–0.75)	Observed: 48 Predicted: NI
Vinkers MT 2008 (pancreas donors) [4]	Pre-procurement pancreas suitability score (pre-transplant to inform donor organ acceptance)	2175 pancreas donors from Eurotransplant database (2002–2005)	Donor pancreas acceptance for transplant	**Donor**: age, BMI, gender, cause of death, cardiac arrest duration, duration of ICU stay, sodium, amylase, lipase, use of vasopressors	Donor age, BMI, duration of ICU stay, duration of cardiac arrest, sodium, amylase/lipase, use of inotropes	Logistic regression model (n = 1)	NI	Observed: 45.3% of grafts declined Predicted: 42.8% risk of P-PASS ≥ 17 and graft declined O/E ratio: 1.06

All graft survival are unadjusted unless specified

*Observed and expected outcomes are for 1-year pancreas survival for PDRI and donor pancreas acceptance for P-PASS (as they are both the outcomes PDRI and P-PASS were derived against respectively) unless otherwise specified

*BMI* body mass index, *CCD* Clinical Consensus Document, *CIT* cold ischaemia time, *CVA* cerebrovascular accident, *DCD* donor after cardiac death, *eGFR* estimated glomerular filtration rate, *H–L* Hosmer–Lemeshow test, *HLA* human leukocyte antigen, *ICU* intensive care unit, *NI* no information, *O/E* observed and predicted (expected) ratio, *p* p value, *PAK* pancreas after kidney transplant, *PDRI* pancreas donor risk index, *P-PASS* pre-procurement pancreas suitability score, *PRA* panel-reactive antibody, *PTA* pancreas transplant alone, *PVD* peripheral vascular disease, *R*^*2*^ coefficient of determination, *SPK* simultaneous pancreas-kidney transplant, *SRTR* Scientific Registry of Transplant Recipients, *UK* United Kingdom, *USA* United States of America

**Table 2:** PDRI and P-PASS predictors

P-PASS (Pre-procurement pancreas suitability score)	PDRI (Pancreas donor risk index)
Age	Age
BMI	BMI
ICU stay (days)	Gender
Cardiac arrest (min)	Asian race
Serum sodium (mmol/L)	Black race
Amylase (U/L) or	Height (cm)
Lipase (U/L)	Cause of death (if CVA/stroke)
(Nor)adrenaline or	CVA/stroke in PAK
Dobuta-/dopamine	Cold ischaemia (h)
	DCD status
	Serum creatinine (if > 2.5 mg/dl)
Donor P-PASS > 17 3 times more likely to be refused	Increased PDRI associated with reduced 1-year pancreas graft survival

*BMI* body mass index, *CIT* cold ischaemia time, *CVA* cerebrovascular accident, *DCD* donor after cardiac death, *ICU* intensive care unit, *PAK* pancreas after kidney transplant, *PDRI* pancreas donor risk index, *P-PASS* pre-procurement pancreas suitability score

### Studies deriving risk indices: model performance

**Fig. 2:**
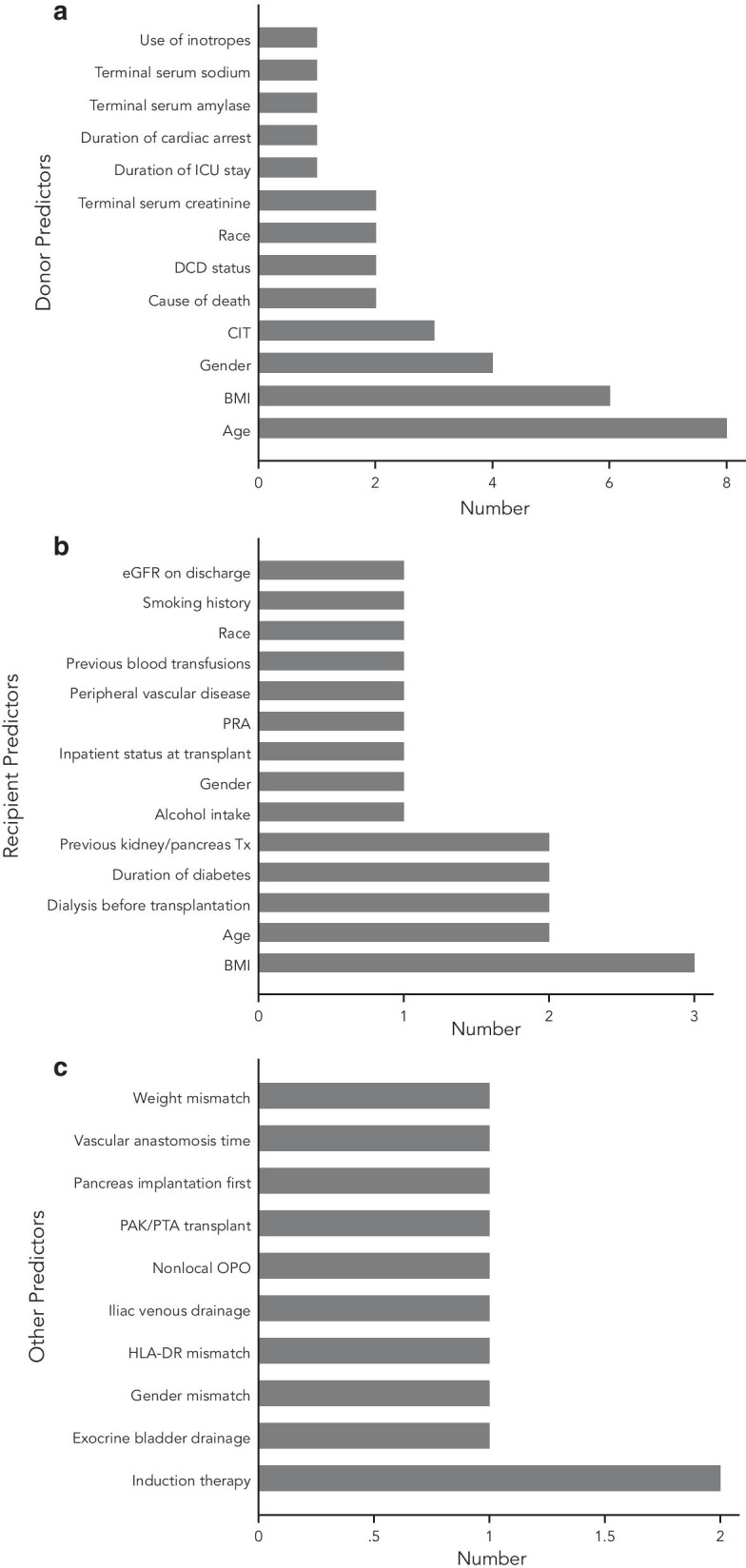
Distribution of donor predictors (**a**), recipient predictors (**b**) and other predictors (**c**) within risk indices derived for use in solid pancreas transplantation

All eight studies deriving risk indices were retrospective. Five studies derived one risk index each [3, 4, 11, 12, 32], two studies derived two risk indices each [13, 35] and one study derived 12 risk indices [33] for outcomes post-solid pancreas transplant (Table 1). In all, 13 donor predictors, 14 recipient predictors and 10 other predictors were used to derive 21 risk indices (Fig. 2a-c). The commonest predictor was donor age (n = 8), followed by donor body mass index (BMI) (n = 6) and donor gender (n = 4).

Discrimination metrics (AUROC/C-statistic) were reported by seven studies (88%) deriving indices (Table 1). Three risk indices predicting 3-month pancreas survival reported C-statistics of 0.52–0.78 [12, 13, 35]. In comparison, five indices (including PDRI) predicting 1-year pancreas survival reported C-statistics of 0.61–0.78 [3, 11, 33]. Five risk indices for 1-year patient survival reported C-statistics of 0.62–0.8 [32, 33, 35]. Six risk indices reported C-statistics of 0.59 to 0.66 for 3-year pancreas survival and 0.64 to 0.76 for 3-year patient survival [33]. The study deriving P-PASS did not report model discrimination for donor pancreas acceptance [4]. Overall, minimal to moderate ability to predict risk was present (where reported) for the studies reporting discrimination metrics.

Model calibration (Hosmer–Lemeshow test or observed/predicted events ratio) was reported by three studies (38%) deriving indices (Table 1). The logistic regression model by Dorsey et al. [13] had a C-statistic of 0.78 for 3-month pancreas survival, with a Hosmer–Lemeshow p value of 0.74. In comparison, the Composite Risk Model [12] had C-statistics of 0.6 to 0.52 for the same outcome depending on the number of risk factors included. The corresponding observed/predicted ratios decreased from 0.8 to 0.2 (with increasing risk factors), with a concurrent decrease in model sensitivity. Meanwhile Kasiske et al. derived 12 models with C-statistics ranging from 0.61 to 0.78 for 1- and 3-year pancreas and graft survival (by transplant type) and reported Hosmer–Lemeshow p values ranging from 0.24 to 0.92 [33]. The PDRI C-statistic was 0.67 for 1-year pancreas survival with no calibration reported [3]. No discrimination was reported for P-PASS however the observed incidence of declining a donor pancreas (45.3%) corresponded to the predicted risk of declining a donor pancreas (42.8%) with a P-PASS ≥ 17 [4].

### Studies deriving risk indices: study quality and risk of bias

Overall study quality of studies deriving risk indices was moderate (Table 1, Additional file 1: Supplement 3). Four studies were in single centre cohorts, three studies used registry data, and one study utilized a multi-centre cohort. Cohorts consisted of recipients of all solid pancreas transplant types (SPK, PAK, PTA) in seven of eight studies deriving risk indices (one study was in an SPK-only cohort [32]). Study outcomes (graft survival) were defined in seven of eight studies, with centre-based reporting of graft survival by one registry-based study [3] (Additional file 1: Supplement 3). Outcomes reported were 3-month pancreas survival (n = 3), 1-year pancreas survival (n = 3), 1-year patient survival (n = 2), 3-year pancreas and patient survival (n = 1) and donor pancreas acceptance (n = 1) (Table 1). Five of eight studies had an events-per-variable (EPV) rate of > 10, lowering the risk of overfitting (Additional file 1: Supplement 3). Missing data was present in one study [11] and was handled via complete case analysis. In three studies, it was unclear whether missing data was present [32, 33, 35]. Four studies had no missing data [3, 4, 12, 13] (Additional file 1: Supplement 3).

Risk indices were modeled differently between studies. The PDRI was developed from significant donor predictors identified via multivariate Cox regression and combined into a continuous risk index with the median donor having a PDRI of 1.0 [3]. In comparison, P-PASS used pre-defined predictors identified by expert opinion to derive logistic regression models for the binary outcomes of donor pancreas acceptance [4]. Initially derived as a continuous score, P-PASS was reclassified into equally-sized categories which were gradually collapsed when regression coefficients were similar, culminating in a model with two categories. Similarly, Dorsey et al. utilized predictors from medical expertise to derive a logistic regression model predicting 3-month pancreas survival. A second model was derived using a backpropagation neural network with all predictors entered as input nodes [13]. Risk indices from other studies were derived using the regression coefficients of significant predictors from multivariate analysis [11, 12, 32, 33, 35]. Only one study (out of eight) derived risk indices by each pancreas transplant type [33]. Only four studies (50%) reported derived risk index equations containing all final predictors with coefficients [3, 11, 12, 32] and only four studies (50%) documented internal validation procedures (Additional file 1: Supplement 3) [3, 4, 12, 33].

The overall PROBAST for studies deriving risk indices was rated at high risk of bias and low applicability (Table 3). All studies except one [13] were at high risk of bias for the ‘Analysis’ domain due to limited reporting of discrimination and calibration metrics. The other PROBAST domains for ‘Participants’, ‘Predictors’ and ‘Outcomes’ had low risk of bias in eight (100%), four (50%) and seven (88%) studies respectively. Four studies (50%) scored poorly for ‘Applicability’ as they included factors that could only be measured at the transplant stage (such as cold ischaemia) or post-transplant stage (such as iliac venous drainage or use of induction therapy), despite being derived for use pre-transplant [12, 32, 33, 35].

**Table 3:** PROBAST assessment for studies deriving risk indices

Study	Risk of Bias				Applicability			Overall	
	Partcipants	Predictors	Outcome	Analysis	Participants	Predictors	Outcome	Risk of bias	Applicability
Axelrod DA 2010 [3]	+	+	−	−	+	−	−	−	+
Dorsey SG 1997 [13]	+	+	+	+	+	+	+	+	+
Finger EB 2013 [12]	+	−	+	−	+	−	+	−	−
Grochowiecki T 2014 [32]	+	−	+	−	+	−	−	−	−
Kasiske BL 2013 [33]	+	−	+	+	+	−	+	−	−
Smigielska 2018 [11]	+	+	+	−	+	+	+	−	+
Sousa M 2014 [35]	+	−	+	−	+	−	+	−	−
Vinkers MT 2008 [4]	+	+	+	−	+	+	+	−	+

*PROBAST* Prediction model Risk of Bias ASsesment Tool, *ROB* risk of bias

+ indicates low ROB/low concern regarding applicability; − indicates high ROB/high concern regarding applicability; ? indicates unclear ROB/unclear concern regarding applicability

### Studies externally validating risk indices: model performance

Of the derived risk indices, only P-PASS and PDRI were further validated in 18 studies (PDRI in 11 studies [8, 9, 11, 12, 36–42] and P-PASS in 14 studies [8, 10, 11, 34, 37–40, 43–47] respectively). However, of these studies, PDRI and P-PASS were validated against their outcomes that they were derived to predict only in 12 studies (PDRI in nine studies [8, 9, 11, 36–39, 42] and P-PASS in three studies [34, 40, 45] respectively) (Table 4). These studies proceeded to undergo study quality and risk of bias assessment. Studies examining PDRI and P-PASS against outcomes for which they were not derived to predict are listed in Additional file 1: Supplement 4.

**Table 4:** Characteristics and results of studies externally validating PDRI and P-PASS

Study, year (transplant type)	Risk index (including any risk groups)	Cohort size, source (study dates)	Outcome	Handling of missing data	Other sources of bias or applicability concerns	Discrimination (or other analysis of association with outcome performed)	Calibration*	Study conclusion
Amaral PHF 2015 (All) [36]	PDRI ≤ 1.0, > 1.0—< 1.5 ≥ 1.5	570 pancreas recipients four hospitals, Brazil (1996–2011)	1-year pancreas survival	Complete case analysis (Only 27% of cohort with PDRI)	No treatment details No comparison of baseline data to derivation study Graft failure undefined Donor race coded differently to derivation Different PDRI cutoffs to derivation (no reason given) Unclear if graft survival was censored	NI	Observed: 23 pancreas failed Predicted: NI	PDRI not associated with 1-year pancreas survival
Ayami MS 2018 (All) [37]	PDRI < 1, 1–1.5, > 1.5	327 pancreas recipients at a single centre, Gernany (2002–2015)	1-, 5-,10-year pancreas, kidney and patient survival	Complete case analysis (Only 98.5% of cohort with PDRI	No treatment details No baseline demographics of predictor variables in cohort No comparison of baseline data to derivation study Different PDRI cutoffs (and not analysed as a continuous model) Donor race set to Caucasian	NI	Observed: 72 pancreas failed Predicted: NI	PDR I > 1.5 associated with worse pancreas survival, but no differences between other risk groups PDRI not associated with kidney or patient survival
Blok JJ 2016 (All) [8]	PDRI < 1.24, ≥ 1.24 (median) and as CN	349 pancreas recipients at a single centre, Germany (1984–2013)	1-, 5-, 10-year pancreas survival (DC)	Complete case analysis (Only 98.6% of cohort with PDRI)	No treatment details Donor race set to Caucasian as not collected by Eurotransplant, No comparison of baseline data to derivation study Different cutoffs for PDRI (median)	PDRI by categories: C-statistic 0.69 (SE 0.045) PDRI as a CN model: NI	Observed: 54 pancreas failed at 1 year Predicted: NI	PDRI ≥ 1.24 associated with worse 1-, 5-, 10-years pancreas survival but not when analyzed as a CN model
Franz C 2019 (SPK, PAK) [38]	PDRI ≥ 1.198 vs < 1.198 (median)	108 pancreas recipients at a single centre, Germany (2000–2017)	1-, 5-year pancreas survival (DC)	NMD	No comparison of baseline data to derivation study PDRI with different cut-offs from original (and not analysed as a continuous model)	NI	Observed: 27 pancreas failed at 1 year Predicted: NI	PDRI ≥ 1.198 vs < 1.198 not associated with all outcomes
Horvath S 2015 (All) [39]	PDRI > 1.57, ≤ 1.57	119 pancreas recipients from single centre, UK (period not clear)	1-year pancreas survival	Complete case analysis (Only 97% of cohort with PDRI)	No treatment details (abstract) Graft failure undefined PDRI with different cutoffs Unclear if graft survival was censored No baseline demographics of predictor variables in cohort	NI	NI	PDRI not associated with 1-year pancreas survival
Kopp W 2016 (pancreas donors) [40]	P-PASS < 17, ≥ 17 and as CN	10,444 pancreas donors from Eurotransplant registry (2004–2014)	Donor pancreas accepted, procured, transplanted	Complete case analysis (Only 93.7% of cohort with P-PASS)	No comparison of baseline data to derivation study Donor race set to Caucasian as not collected routinely	C-statistic for P-PASS in donors: Reported vs non-reported (0.63, 95% CI 0.62–0.63) Accepted vs non-accepted (0.68, 95% CI 0.67–0.69) Procured vs non-procured (0.68, 95% CI 0.67–0.69) Transplanted vs non-transplanted (0.73, 95% CI 0.72–0.74)	Observed: 4354 (41.7%) donor pancreas declined Predicted: 6298 (60.3%) donors with P-PASS ≥ 17 O/E ratio: 0.69	P-PASS < 17 associated with reported, accepted, procured and transplanted pancreas donors vs P-PASS ≥ 17
Lan L 2010 (pancreas donors) [45]	Modified P-PASS (minus serum sodium) (CN)	220 pancreas donors from a single centre, Australia (2005–2008)	Donor pancreas acceptance	NI	No treatment details (abstract) Serum sodium not included as not collected by registry (different to derivation) No comparison of baseline data to derivation study	NI	Observed: 93 donor pancreas declined Predicted: NI	Modified P-PASS associated with pancreas donor acceptance
Mittal S 2013 (SPK and PTA) [41]	PDRI 0.64–0.85 0.86–1.15 1.16–1.56 1.57–2.11 2.11–2.56 > 2.57	90 pancreas recipients from a single centre, UK (2011)	1-year pancreas survival	NMD	Graft failure undefined No treatment details	NI	O/E ratio: 0.84–1.1 (SPK) 0.76–1.12 (PTA)	Higher PDRI quintiles associated with 1-year pancreas survival for SPK but not PTA
Mittal S 2015 (All) [42]	PDRI (CN)	1265 pancreas recipients from a national registry, UK (2004–2011)	1-year pancreas survival (DC)	Complete case analysis (Only 76% of cohort with PDRI)	None	NI	Observed: NI Predicted: NI	PDRI associated with 1-year pancreas survival for SPK
	PDRI 0.64–0.85 0.86–1.15 1.16–1.56 1.57–2.11					NI	NI	PDRI quartile 0.64–0.85 associated with better 1-year pancreas survival for SPK vs quartiles 1.16–1.56, 1.57–2.11
Rodriguez-Villar C 2018 (Pancreas donors) [34]	P-PASS < 17, ≥ 17	78 pancreas donors at a single centre, Spain (2016–2017)	Donor pancreas acceptance	NMD	No comparison of baseline data to derivation study	NI	Observed: 41 donor pancreas declined Predicted: 57 donor pancreas declined O/E ratio: 0.72	P-PASS associated with donor pancreas acceptance
Salamanca-Bustos JJ 2016 (SPK) [9]	PDRI 0.64–0.85 0.86–1.15 1.16–1.56 1.57–2.11 2.12–2.86	126 pancreas recipients at a single-centre, Spain (2000–2015)	1-year pancreas survival	Complete case analysis (Only 94% of cohort with PDRI)	All donors set to Caucasian No treatment details No comparison of baseline data to derivation study	NI	Observed: 16 pancreas failure Predicted: NI	PDRI in quintiles not associated with 1-year pancreas survival
Smigielska K 2018 (All) [11]	PDRI (CN)	408 pancreas recipients at multiple centres, Poland (1998–2015)	1-year pancreas survival	Complete case analysis (Only 73% of cohort with PDRI)	No treatment details No comparison of baseline data to derivation study	AUROC for PDRI: 0.524	Observed: 139 pancreas failure Predicted: NI	PDRI not associated with 1-year pancreas

All graft survival is uncensored for death unless otherwise stated

*Observed and expected outcomes are for 1-year pancreas survival for PDRI and donor pancreas acceptance for P-PASS (both the outcomes PDRI and P-PASS were derived against respectively) unless otherwise specified

**Based on mean/median PDRI for entire cohort

*A* abstract, *CIT* cold ischaemia time, *CN* continuous, *DC* death-censored (all studies non-DC unless stated), *DP* donor pancreas, *F* full-text, *MC* multicentre, *NI* no information, *NMD* no missing data, *O/E* observed and predicted (expected) ratio, *PDRI* pancreas donor risk index, *PGS* pancreas graft survival, *P-PASS* pre-procurement pancreas allocation suitability score, *PTA* pancreas transplant alone, *R* registry, *SC* single centre, *SE* standard error, *UK* United Kingdom, *USA* United States of America

Only two of the 12 studies (17%) externally validating PDRI reported discrimination metrics. Blok et al. reported a C-statistic of 0.69 for PDRI for an association between PDRI and pancreas survival up to 10 years when a cut-off of 1.24 was used [8]. This was similar to the C-statistic reported in the PDRI derivation study. Smigielska et al. reported a AUROC of 0.52 for PDRI as a continuous model in predicting 1-year pancreas survival but found that PDRI was not associated with the outcome [11]. Only one of 12 studies (8%) validating PDRI reported observed/expected ratios for calibration (ranging from 0.76 to 1.12 by quintile and transplant type) [41] however the study deriving PDRI [3] did not report calibration metrics hence no comparison was possible.

Of the four studies utilizing PDRI by quintiles (as per its’ derivation), two reported an association between PDRI and pancreas survival (only in SPK transplants from two studies) [41, 42]. Of the three studies utilizing PDRI as a continuous model, one study reported an association between PDRI and 1-year pancreas survival [42]. From the five studies validating PDRI via different risk groups to derivation, only one study reported an association with pancreas survival [8] suggesting that reclassification of PDRI during external validation may have affected the outcome.

For P-PASS, one of three (33%) external validation studies reported discrimination and calibration metrics. Kopp et al. reported a C-statistic of 0.68 for P-PASS and the outcome of donor pancreas acceptance [40]. However, observed/exposed ratios of 0.69 to 0.72 were reported by two studies (67%) [34, 40] compared to P-PASS derivation (observed/exposed ratio 1.06) [4]. All three studies validating P-PASS for donor pancreas acceptance reported an association with the outcome [34, 40, 45].

### Studies externally validating risk indices: study quality and risk of bias

Overall study quality of the external validation studies was poor (Table 4). Of the 12 external validation studies, eight studies utilized single-centre cohorts, two studies were registry-based, and one study utilized a multi-centre cohort. Missing data was present in eight studies (62%), varying from 1.4 to 73% of the cohort [8, 9, 11, 36, 37, 39, 40, 42]. This was handled by complete case analysis in all eight studies. Three studies had no missing data [34, 38, 41] and one study did not report missing data [45]. Graft failure was not defined in three studies (23%) [36, 39, 45]. Model predictors were reclassified in several studies. In five studies, PDRI was categorised differently to how it was originally derived [8, 36–39]. In these studies, PDRI was classified either as ‘high’ or ‘low’ according to the median PDRI within the cohort, or in tertiles. Furthermore, donor race was classified differently to that of PDRI derivation in one study due to differences in that country [36] and was not clearly classified in two studies [11, 38] (not reported with other study variables). P-PASS was also validated while omitting serum sodium in one study due to lack of reporting in that jurisdiction [45].

Due to the limited reporting of discrimination and calibration metrics, risk of bias for the ‘Analysis’ domain in PROBAST was high in all but one study [40] (Table 5). However, domains for ‘Participants’, ‘Predictors’ recorded low risk of bias for all 12 studies, while ‘Outcomes’ had low risk of bias in nine studies (75%). ‘Applicability’ was rated low for two studies(17%) [36, 45] due to predictors being modified as previously described, and unclear for four studies [11, 38, 39, 41] due to lack of information on outcome definition and predictor collection.

**Table 5:** PROBAST risk of bias assessment for studies externally validating PDRI/P-PASS

Study	Risk of bias				Applicability			Overall	
	Participants	Predictors	Outcome	Analysis	Participants	Predictors	Outcome	Risk of bias	Applicability
Amaral PHF 2015 [36]	+	+	?	−	+	−	?	−	−
Ayami M 2016 [37]	+	+	+	−	+	+	+	−	+
Blok JJ 2016 [8]	+	+	+	−	+	+	+	−	+
Franz C 2019 [38]	+	+	+	−	+	?	+	−	?
Horvath S 2015 [39]	+	+	−	−	+	?	?	−	−
Kopp W 2016 [40]	+	+	+	+	+	+	+	+	+
Lan L 2010 [45]	+	+	+	−	+	−	+	−	−
Mittal S 2013 [41]	+	+	?	−	+	+	?	−	?
Mittal S 2015 [42]	+	+	+	−	+	+	+	−	+
Rodriguez-Villar C 2018 [34]	+	+	+	−	+	+	+	−	+
Salamanca-Bustos JJ 2016 [9]	+	+	+	−	+	+	+	−	+
Smigielska K 2018 [11]	+	+	+	−	+	?	+	−	?

*PROBAST* Prediction model Risk of Bias ASsesment Tool, *ROB* risk of bias

+ indicates low ROB/low concern regarding applicability; − indicates high ROB/high concern regarding applicability;? indicates unclear ROB/unclear concern regarding applicability

### GRADE assessment

All derivation and external validation studies were included in the GRADE assessment of the overall quality of evidence by outcomes. Baseline evidence quality was downgraded to ‘Moderate’ as all studies were retrospective and non-randomised [30]. This was further downgraded to ‘Low’ due to the high risk of bias as per PROBAST. For studies examining PDRI as a continuous score as well as via various risk categories, GRADE was downgraded for ‘Inconsistency’ domain as varying degrees of association with the outcome were present. For studies validating PDRI using different PDRI risk groups (to its’ derivation) GRADE was downgraded for the ‘Indirectness’ domain. Some outcomes included only one or two studies or were performed in small cohorts with low event rates, thus GRADE was downgraded for ‘Imprecision’ (Table 6).

**Table 6:** GRADE assessment of included outcomes for PDRI and P-PASS

Outcome	Risk index	Studies^	GRADE quality	Footnotes*	Interpretation
1-year pancreas survival	PDRI as a continuous score	4	⊕ VERY LOW	2 studies demonstrated an association with outcome Downgraded for: Outcomes differing between studies (*INCONSISTENCY*)	Very low quality evidence that PDRI as a continuous variable is associated with 1-year pancreas survival
	PDRI as quintiles	4	⊕⊕ LOW	3 of 4 studies demonstrated an association with the outcome Association with SPK transplants in 2 studies	Low quality evidence that PDRI in quartiles is associated with 1-year pancreas survival (particularly SPK transplants)
	PDRI as categorical model (various risk groups)	5	⊕ VERY LOW	1 of 5 studies demonstrated an association with outcome Downgraded for: Differing PDRI risk groups (*INDIRECTNESS*) Outcomes differing between studies (*INCONSISTENCY*) Small total event rate < 300 (*IMPRECISION*)	Very low quality evidence that PDRI as a categorical model (by various risk groups) is associated with 1-year pancreas survival
1-year patient survival	PDRI as tertiles	1	⊕ VERY LOW	No association with outcome Downgraded for: Small number of studies (*IMPRECISION*)	Very low quality evidence that PDRI by tertiles is associated with 1-year patient survival
Donor pancreas acceptance	P-PASS < 17vs ≥ 17	4	⊕⊕ LOW	All studies demonstrated an association with outcome	Low quality evidence that P-PASS is associated with donor pancreas acceptance

*No pooling of discrimination metrics or other effect estimates unless otherwise stated. All outcome evidence was downgraded at baseline due to the observational nature of the studies as well as their risk of bias

*PDRI* pancreas donor risk index, *P-PASS* pre-procurement pancreas suitability score, *SPK* simultaneous pancreas-kidney transplant

In summary, ‘Low’ quality evidence exists for PDRI (as quintiles per derivation) in predicting risk of 1-year pancreas survival and for P-PASS in predicting donor pancreas acceptance (Table 6). Even less evidence exists in utilizing PDRI by different risk strata to its’ derivation, and for PDRI in predicting 1-year patient survival.

## Discussion

This systematic review of risk indices derived for use in solid pancreas transplantation found that despite 21 risk indices being derived, only P-PASS and PDRI were externally validated and are in use today [6, 7]. PDRI (derived in USA) was validated in a UK cohort [41, 42] while P-PASS (derived in the Netherlands) was validated in Spanish [34] and Australian [45] cohorts, albeit in a modified form in the latter.

PDRI discrimination for 1-year pancreas survival was poor to moderate (C-statistic/AUROC 0.52–0.69) and P-PASS discrimination was moderate for donor pancreas acceptance (C-statistic 0.68). Calibration was poorly reported in both PDRI and P-PASS derivation and external validation studies. In P-PASS external validation studies, calibration (in form of O/E ratios) was lower than in its’ derivation, suggesting a degree of overestimation. A contributing factor to this from one study was that donor pancreas acceptance in that particular cohort was determined by more liberal donor pancreas acceptance cut-offs compared to the P- PASS derivation study [34].

Within the studies included in our review, discrimination and calibration metrics were only reported in 88% and 38% of risk index derivation studies, 17% and 8% of studies externally validating PDRI and 33% and 67% of studies externally validating P-PASS respectively. This limits the applicability of such indices in other cohorts external to its’ derivation. Limited reporting of these metrics in prediction modelling has been previously reported in a systematic review of clinical prediction studies in 2008, where discrimination and calibration were only reported in 27% and 12% of studies respectively [48]. This led to the introduction of TRIPOD to ensure completeness of data reporting in prediction studies [16]. A review of studies published before TRIPOD’s inception found incomplete information to guide use of prediction models was present in > 80% of derived models [49], an issue also present in our review (Additional file 1: Supplement 2).

Our review also identified 13 studies analysing the association of PDRI (n = 2) and P-PASS (n = 11) for outcomes they were not derived to predict (Additional file 1: Supplement 4). P-PASS was analysed for an association with pancreas survival in 11 studies [8, 10, 11, 37–39, 43, 44, 46, 47, 50] (no significant association in eight studies). PDRI was associated with graft survival at 3 months in one study [12], and associated with donor pancreas acceptance in another study [40]. Therefore P-PASS in particular should not be utilised to predict pancreas survival outcomes. Our review also demonstrates that using PDRI with different risk categories to that of its’ derivation, or in different cohorts without proper reclassification measures reduces its’ predictive ability for pancreas survival.

Elsewhere, PDRI and P-PASS have been analysed along with other independent variables for associations with post-transplant outcomes. PDRI was associated with 1-year pancreas and patient survival in a study examining the correlation of immunological matching with graft rejection and survival in pancreas transplantation [51]. P-PASS however was not associated with pancreas survival in a study analysing donor and recipient factors predicting graft survival post-pancreas transplantation [52], again correlating with our finding of P-PASS being poorly associated with pancreas graft survival as it was not derived to predict this.

A study limitation was the inclusion of abstracts as we anticipated a limited number of studies meeting our inclusion criteria. To counter this, we contacted study authors to obtain supporting information for any abstracts meeting our inclusion criteria however received few responses. Also, the use of C-statistic/AUROC as a means of assessing discrimination has been discussed elsewhere as these metrics do not account for study heterogeneity and the predicted probabilities of individual variables upon the outcome [53]. Notwithstanding this, the TRIPOD assessment of discrimination includes the C-statistic/AUROC hence we have considered it an acceptable metric for this purpose. Finally, as risk indices are used in combination with clinical judgement to make transplantation decisions, other factors may confound the results. Studies externally validating risk indices should compare their baseline cohort characteristics to that of the risk index derivation study, as well as other factors such as the immunosuppression regimen used in order to stratify for key differences. Unfortunately, comparisons of baseline study characteristics were not made in 75% of external validation studies and immunosuppression regimen were not detailed in 67% of external validation studies (Table 4).

Beyond the limited number of quality external validation studies, P-PASS and PDRI have other factors limiting their use in other cohorts. While able to predict which donor pancreata should be accepted, P-PASS was not derived to predict post-transplant outcomes [4] hence limiting its’ ability to meaningfully guide pancreas transplantation decisions. While PDRI is associated with pancreas graft survival, it’s predictive ability is best at differentiating risk between extreme PDRI values [3], thus PDRI values close to the median are less easy to interpret. Furthermore in some cohorts, PDRI has only been able to predict graft survival for SPK transplantation (as opposed to PAK or PTA transplants) [42].

Current solid pancreas transplant protocols identify suitable pancreas donors without established high-risk factors (i.e. cause of death from trauma, age below 40–50 years old, BMI under 30 kg/m^2^ and cold ischaemic time (CIT) below 12 h) while donors beyond such criteria are either not accepted or allocated to islet cell transplantation [54–56]. However, such an approach may lead to an under-utilisation of donor pancreata which do not meet all the above criteria. Validated indices taking into account donor factors at time of donor offer and estimating graft or patient survival could aid in decisions for transplantation, particularly for donors who have borderline criteria by current standards. Also, incorporating recipient and other risk factors (also present at time of donor offer) [33, 35] in future risk indices may further improve their predictive ability for post-transplant outcomes. Similar risk indices (with discriminatory metrics similar to that of PDRI) incorporating both donor and recipient covariates are currently being used to guide kidney transplantation decisions both locally and abroad [57, 58].

Currently in Australia and New Zealand, donor race is coded differently to that of the PDRI and donor serum sodium is not routinely collected by the Australia and New Zealand Pancreas Transplant Registry. Therefore, to validate PDRI or P-PASS for use locally would require reclassification measures. Furthermore, CIT as a covariate in PDRI is not usually available at time of donor offer. Axelrod et al. acknowledge this and suggest setting the CIT to 12 h (the reference value) in such cases [3]. A similar approach would be taken with other variables such as donor ethnicity. An alternative approach is to retrospectively review local data present at time of organ offer to identify significant donor and recipient covariates associated with pancreas transplant outcomes to derive and validate a risk index which could guide local donor pancreas acceptance decisions.

## Conclusions

Current data quality of studies deriving and externally validating risk indices for use in solid pancreas transplantation is inadequate. External validation for 90% of derived risk indices for solid pancreas transplantation was not performed. PDRI and P-PASS are the only risk indices currently externally validated for use in solid pancreas transplantation. PDRI was derived and validated for the outcomes of 1-year pancreas survival while P-PASS was derived and validated for donor pancreas acceptance for transplantation. Due to inadequate reporting of model performance metrics, there is currently low evidence to support their use outside current externally validated cohorts, or with different cut-offs to their derivation. To validate either risk index for use in Australia/New Zealand would require reclassification measures due to differences in covariate coding. However, incorporating recipient and other factors which are associated with post-transplant outcomes alongside current donor covariates such as those within PDRI may increase predictive ability for future risk indices to guide solid pancreas transplantation decisions.

## Supplementary Information

**Additional file 1**. Supplementary Files.

## Data Availability

The datasets used and/or analysed during the study are available from the corresponding author on reasonable request.

## Abbreviations

AUROC: Area under the receiver operating characteristic curve
BMI: Body mass index
CHARMS: Critical Appraisal and Data Extraction for Systematic Reviews of Prediction Modelling Studies
CCD: Clinical Consensus Document
CIT: Cold ischaemic time
CVA: Cerebrovascular accident
DBD: Donation after brain death
DCD: Donation after cardiac death
EDC: Extended donor criteria
eGFR: Estimated glomerular filtration rate
EPV: Events per variable
GRADE: Grading of Recommendations, Assessment, Development and Evaluations framework
H–L: Hosmer–Lemeshow test
HLA: Human leukocyte antigen
ICU: Intensive care unit
MESH: Medical subject headings
NP: Not performed
P: P value
PAK: Pancreas after kidney transplant
PDRI: Pancreas Donor Risk Index
P-PASS: Pre-procurement Pancreas Allocation Suitability Score
PRA: Panel-reactive antibody
PRISMA: Preferred Reporting Items for Systematic Reviews and Meta-Analyses
PROBAST: Prediction Model Risk of Bias Assessment Tool
PTA: Pancreas transplant alone
PVD: Peripheral vascular disease
R^2^: Co-efficient of determination
ROC: Receiver operating curve
SPK: Simultaneous pancreas kidney transplantation
SRTR: The Scientific Registry of Transplant Recipients
T1DM: Type 1 diabetes mellitus
T2DM: Type 2 diabetes mellitus
TRIPOD: Transparent Reporting of a multivariable prediction model for Individual Prognosis or Diagnosis
UK: United Kingdom
USA: United States of America
