# Supplementary material for: Risk indices predicting graft use, graft and patient survival in solid pancreas transplantation: a systematic review
Source: BMC Gastroenterol. 2021 Feb 23;21:80. doi: 10.1186/s12876-021-01655-2 (PMC7901078; doi:10.1186/s12876-021-01655-2)
Supplement: Supplementary file 1 — Additional file 1. Supplementary Files. [file 12876_2021_1655_MOESM1_ESM.docx]

Risk Indices Predicting Graft use, Graft and Patient Survival in Solid Pancreas Transplantation: A Systematic Review

**Authors**:

1. Jonathan EH Ling^1.2^, FRACP
2. Timothy Coughlan^3^, FRACP
3. Kevan R Polkinghorne^1,2,4^, Professor
4. John Kanellis^1.2^, Professor

**Affiliations**:

1. Department of Nephrology, Monash Medical Centre, Monash Health, Clayton, Melbourne, Australia

2. Centre for Inflammatory Diseases, Department of Medicine, Monash University, Clayton, Melbourne, Australia

3. Department of Renal Services, Latrobe Regional Hospital, Victoria, Australia

4. Department of Epidemiology and Preventive Medicine, School of Public Health and Preventive Medicine, Monash University, Prahran, Melbourne, Australia

**Address for correspondence**:

Jonathan Ling,

Department of Nephrology, Monash Medical Centre,

246 Clayton Road, Clayton 3168,

Victoria, Australia

Tel: +6195943520, Fax: +6195946530, Email: jlingeh@gmail.com

**Clinical Trial Notation**: Not applicable

**Supplement 1: Search protocols**

COCHRANE

ID Search

#1 MeSH descriptor: [Pancreas Transplantation] explode all trees

#2 SPK

#3 simultaneous kidney pancreas near/1 transplant*

#4 simultaneous pancreas kidney near/1 transplant*

#5 "pancreas after kidney"

#6 (transplant* or graft* or allocation or donor* or selection*) near/1 pancrea*

#7 MeSH descriptor: [Risk Assessment] this term only

#8 score*

#9 "scoring system*"

#10 index or indices

#11 model or models

#12 PDRI or "pancreas donor risk index"

#13 P-PASS or "Preprocurement pancreas allocation suitability score"

#14 tool or tools

#15 #1 or #2 or #3 or #4 or #5 or #6

#16 #7 or #8 or #9 or #10 or #11 or #12 or #13 or #14

#17 #15 and #16

Embase via Ovid

1. exp pancreas transplantation/
2. SPK.mp. [mp=title, abstract, heading word, drug trade name, original title, device manufacturer, drug manufacturer, device trade name, keyword, floating subheading word]
3. (simultaneous kidney pancreas adj2 transplant^).mp. [mp=title, abstract, heading word, drug trade name, original title, device manufacturer, drug manufacturer, device trade name, keyword, floating subheading word]
4. (simultaneous pancreas kidney adj2 transplant^).mp. [mp=title, abstract, heading word, drug trade name, original title, device manufacturer, drug manufacturer, device trade name, keyword, floating subheading word]
5. pancreas after kidney.mp. [mp=title, abstract, heading word, drug trade name, original title, device manufacturer, drug manufacturer, device trade name, keyword, floating subheading word]
6. ((transplant^ or graft^ or allocation or donor^ or selection) adj1 pancrea^).mp. [mp=title, abstract, heading word, drug trade name, original title, device manufacturer, drug manufacturer, device trade name, keyword, floating subheading word]
7. 1 or 2 or 3 or 4 or 5 or 6
8. 2 or 3 or 4 or 5 or 6
9. (SPK or (simultaneous kidney pancreas adj2 transplant^) or (simultaneous pancreas kidney adj2 transplant^) or pancreas after kidney or ((transplant^ or graft^ or allocation or donor^ or selection) adj1 pancrea^)).ti,ab.
10. 1 or 9
11. risk assessment/
12. score^.mp. [mp=title, abstract, heading word, drug trade name, original title, device manufacturer, drug manufacturer, device trade name, keyword, floating subheading word]
13. scoring system^.mp. [mp=title, abstract, heading word, drug trade name, original title, device manufacturer, drug manufacturer, device trade name, keyword, floating subheading word]
14. (index or indices).mp. [mp=title, abstract, heading word, drug trade name, original title, device manufacturer, drug manufacturer, device trade name, keyword, floating subheading word]
15. (model or models).mp. [mp=title, abstract, heading word, drug trade name, original title, device manufacturer, drug manufacturer, device trade name, keyword, floating subheading word]
16. (tool or tools).mp. [mp=title, abstract, heading word, drug trade name, original title, device manufacturer, drug manufacturer, device trade name, keyword, floating subheading word]
17. (PDRI or Pancreas donor risk index).mp. [mp=title, abstract, heading word, drug trade name, original title, device manufacturer, drug manufacturer, device trade name, keyword, floating subheading word]
18. (P-PASS or preprocurement pancreas allocation suitability score).mp. [mp=title, abstract, heading word, drug trade name, original title, device manufacturer, drug manufacturer, device trade name, keyword, floating subheading word]
19. 11 or 12 or 13 or 14 or 15 or 16 or 17 or 18
20. 12 or 13 or 14 or 15 or 16 or 17 or 18
21. (score^ or scoring system^ or (index or indices) or (model or models) or (tool or tools) or (PDRI or Pancreas donor risk index) or (P-PASS or preprocurement pancreas allocation suitability score)).ti,ab
22. 11 or 21
23. 7 and 19
24. 10 and 22

Ovid MEDLINE Plus

1. Pancreas Transplantation/
2. SPK.mp. [mp=title, abstract, name of substance word, subject heading word, keyword heading word, protocol supplementary concept word, rare disease supplementary concept word, unique identifier synonyms]
3. (simultaneous kidney pancreas adj2 transplant^).mp. [mp=title, abstract, name of substance word, subject heading word, keyword heading word, protocol supplementary concept word, rare disease supplementary concept word, unique identifier synonyms]
4. (simultaneous pancreas kidney adj2 transplant^).mp. [mp=title, abstract, name of substance word, subject heading word, keyword heading word, protocol supplementary concept word, rare disease supplementary concept word, unique identifier synonyms]
5. pancreas after kidney.mp. [mp=title, abstract, name of substance word, subject heading word, keyword heading word, protocol supplementary concept word, rare disease supplementary concept word, unique identifier synonyms]
6. ((transplant^ or graft^ or allocation or donor^ or selection) adj1 pancrea^).mp. [mp=title, abstract, name of substance word, subject heading word, keyword heading word, protocol supplementary concept word, rare disease supplementary concept word, unique identifier synonyms]
7. 1 or 2 or 3 or 4 or 5 or 6
8. 2 or 3 or 4 or 5 or 6
9. (SPK or (simultaneous kidney pancreas adj2 transplant^) or (simultaneous pancreas kidney adj2 transplant^) or pancreas after kidney or ((transplant^ or graft^ or allocation or donor^ or selection) adj1 pancrea^)).ti,ab.
10. 1 or 9
11. Risk Assessment/
12. score^.mp. [mp=title, abstract, name of substance word, subject heading word, keyword heading word, protocol supplementary concept word, rare disease supplementary concept word, unique identifier synonyms]
13. scoring system^.mp. [mp=title, abstract, name of substance word, subject heading word, keyword heading word, protocol supplementary concept word, rare disease supplementary concept word, unique identifier synonyms]
14. (index or indices).mp. [mp=title, abstract, name of substance word, subject heading word, keyword heading word, protocol supplementary concept word, rare disease supplementary concept word, unique identifier synonyms]
15. (model or models).mp. [mp=title, abstract, name of substance word, subject heading word, keyword heading word, protocol supplementary concept word, rare disease supplementary concept word, unique identifier synonyms]
16. (tool or tools).mp. [mp=title, abstract, name of substance word, subject heading word, keyword heading word, protocol supplementary concept word, rare disease supplementary concept word, unique identifier synonyms]
17. (PDRI or Pancreas donor risk index).mp. [mp=title, abstract, name of substance word, subject heading word, keyword heading word, protocol supplementary concept word, rare disease supplementary concept word, unique identifier synonyms]
18. (P-PASS or preprocurement pancreas allocation suitability score).mp. [mp=title, abstract, name of substance word, subject heading word, keyword heading word, protocol supplementary concept word, rare disease supplementary concept word, unique identifier synonyms]
19. 11 or 12 or 13 or 14 or 15 or 16 or 17 or 18
20. 12 or 13 or 14 or 15 or 16 or 17 or 18
21. (score^ or scoring system^ or (index or indices) or (model or models) or (tool or tools) or (PDRI or Pancreas donor risk index) or (P-PASS or preprocurement pancreas allocation suitability score)).ti,ab.
22. 11 or 21
23. 7 and 19
24. 10 and 22

Grey literature search (Web of Science, Scopus, OpenGrey)

( TITLE-ABS-KEY ( pancreas  AND transplant ) )  AND  ( TITLE-ABS-KEY ( risk  AND index )  OR  TITLE-ABS-KEY ( model  OR score ) )


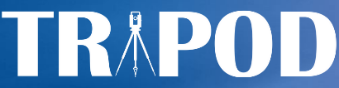


**Supplement 2: TRIPOD checklist**^16^ **for prediction model development and validation for risk indices in solid pancreas transplantation**

| **Section/Topic** |  | **Item** |  | **Checklist Item** |  |  |
| --- | --- | --- | --- | --- | --- | --- |
| **Title and abstract** | | | | | **Total applicable studies, N** | **Adherent studies, N (%)** |
| Title |  | 1 | D;V | Identify the study as developing and/or validating a multivariable prediction model, the target population, and the outcome to be predicted. | 26 | 6 (23) |
| Abstract |  | 2 | D;V | Provide a summary of objectives, study design, setting, participants, sample size, predictors, outcome, statistical analysis, results, and conclusions. | 26 | 1 (4) |
| **Introduction** | | | | | |  |
| Background and objectives |  | 3a | D;V | Explain the medical context (including whether diagnostic or prognostic) and rationale for developing or validating the multivariable prediction model, including references to existing models. | 26 | 22 (85) |
|  |  | 3b | D;V | Specify the objectives, including whether the study describes the development or validation of the model or both. | 26 | 21 (81) |
| **Methods** | | | | | |  |
| Source of data |  | 4a | D;V | Describe the study design or source of data (e.g., randomized trial, cohort, or registry data), separately for the development and validation data sets, if applicable. | 26 | 26 (100) |
|  |  | 4b | D;V | Specify the key study dates, including start of accrual; end of accrual; and, if applicable, end of follow-up. | 26 | 20 (77) |
| Participants |  | 5a | D;V | Specify key elements of the study setting (e.g., primary care, secondary care, general population) including number and location of centres. | 26 | 24 (92) |
|  |  | 5b | D;V | Describe eligibility criteria for participants. | 26 | 26 (100) |
|  |  | 5c | D;V | Give details of treatments received, if relevant. | 20 | 8 (40) |
| Outcome |  | 6a | D;V | Clearly define the outcome that is predicted by the prediction model, including how and when assessed. | 26 | 18 (69) |
|  |  | 6b | D;V | Report any actions to blind assessment of the outcome to be predicted. | 26 | 25 (96) |
| Predictors |  | 7a | D;V | Clearly define all predictors used in developing or validating the multivariable prediction model, including how and when they were measured. | 26 | 16 (62) |
|  |  | 7b | D;V | Report any actions to blind assessment of predictors for the outcome and other predictors. | 26 | 26 (100) |
| Sample size |  | 8 | D;V | Explain how the study size was arrived at. | 26 | 26 (100) |
| Missing data |  | 9 | D;V | Describe how missing data were handled (e.g., complete-case analysis, single imputation, multiple imputation) with details of any imputation method. | 26 | 19 (73) |
| Statistical analysis methods |  | 10a | D | Describe how predictors were handled in the analyses. | 8 | 2 (25) |
|  |  | 10b | D | Specify type of model, all model-building procedures (including any predictor selection), and method for internal validation. | 8 | 1 (13) |
|  |  | 10c | V | For validation, describe how the predictions were calculated. | 18 | 14 (78) |
|  |  | 10d | D;V | Specify all measures used to assess model performance and, if relevant, to compare multiple models. | 26 | 2 (8) |
|  |  | 10e | V | Describe any model updating (e.g., recalibration) arising from the validation, if done. | 1 | 1 (100) |
| Risk groups |  | 11 | D;V | Provide details on how risk groups were created, if done. | 17 | 15 (88) |
| Development vs. validation |  | 12 | V | For validation, identify any differences from the development data in setting, eligibility criteria, outcome, and predictors. | 18 | 14 (78) |
| **Results** | | | | | |  |
| Participants |  | 13a | D;V | Describe the flow of participants through the study, including the number of participants with and without the outcome and, if applicable, a summary of the follow-up time. A diagram may be helpful. | 26 | 24 (92) |
|  |  | 13b | D;V | Describe the characteristics of the participants (basic demographics, clinical features, available predictors), including the number of participants with missing data for predictors and outcome. | 26 | 15 (58) |
|  |  | 13c | V | For validation, show a comparison with the development data of the distribution of important variables (demographics, predictors and outcome). | 18 | 1 () |
| Model development |  | 14a | D | Specify the number of participants and outcome events in each analysis. | 8 | 8 (100) |
|  |  | 14b | D | If done, report the unadjusted association between each candidate predictor and outcome. | 6 | 6 (100) |
| Model specification |  | 15a | D | Present the full prediction model to allow predictions for individuals (i.e., all regression coefficients, and model intercept or baseline survival at a given time point). | 8 | 4 (50) |
|  |  | 15b | D | Explain how to the use the prediction model. | 8 | 2 (25) |
| Model performance |  | 16 | D;V | Report performance measures (with CIs) for the prediction model. | 26 | 2 (8) |
| Model-updating |  | 17 | V | If done, report the results from any model updating (i.e., model specification, model performance). | 1 | 1 (100) |
| **Discussion** | | | | | |  |
| Limitations |  | 18 | D;V | Discuss any limitations of the study (such as nonrepresentative sample, few events per predictor, missing data). | 26 | 23 (88) |
| Interpretation |  | 19a | V | For validation, discuss the results with reference to performance in the development data, and any other validation data. | 18 | 15 (83) |

**Supplement 3: Study quality of studies deriving risk indices**

| **Study (year)** | **Risk index** | **Outcome definition (Y/N)** | **Handling of missing data** | **Events per variable** | **Internal validation measures** | **Other sources of bias/ applicability concerns** |
| --- | --- | --- | --- | --- | --- | --- |
| Axelrod D 2010 ^3^ | PDRI | Defined by centre reporting | NMD | 61.4 | Split-sampling using most recent 2.5 years of cohort | No standardized definition of pancreas survival (center-based reporting)  CIT may not be available at time of organ offer  Model variables and co-efficients present |
| Dorsey SG 1997 ^13^ | Logistic regression model and a neural network model | Y | NMD | 1.92 | None | Model variables present but not co-efficients |
| Finger EB 2013 ^12^ | Composite Risk Index | Y | NMD | 4.73 | Bootstrap resampling of the entire cohort (1000 times) | Model variables and co-efficients present |
| Grochowiecki T 2014 ^32^ | Logistic regression model | N | ND | 4.1 | None | Model variables and co-efficients present |
| Kasiske BL 2013 ^33^ | 12 models via Cox regression | Y | ND | 2.95 to 20 (based on transplant type and outcome) | Split-sampling | Registry data, therefore data entered dependent on centre  CIT and HLA mismatch not known at time of offer  Model variables present but not co-efficients |
| Smigielska K 2018 ^11^ | Logistic regression model | Y | CCA (Missing data for 1 patient) | 14.64 | None | Model variables and co-efficients present |
| Sousa M 2014 ^35^ | Two Cox regression models | Y | ND | 14.67 | None | Presence of induction therapy is not a factor present at time of organ offer  Model variables and co-efficients present |
| Vinkers MT 2008 ^48^ | P-PASS | Y | NMD | 130 | Validated on entire cohort | Model variables present but not co-efficients |

*CAT: analyzed as categorical, CCA: complete case analysis, CIT: cold ischaemic time, CN: analyzed as continuous, HLA: human leukocyte antigen, MC: multicentre, N/a: not applicable, ND: not documented, NMD: no missing data, PDRI: pancreas donor risk index, PGS: pancreas graft survival, P-PASS: pre-procurement pancreas allocation suitability score, PS: patient survival, R: registry, SC: single centre*

**Supplement 4: Studies externally validating PDRI and P-PASS for other outcomes (other than they were derived for).**

| **Study, year (transplant type)** | **Risk index (including any risk groups)** | **Cohort size, source (study dates)** | **Outcome** | **Handling of missing data** | **Other sources of bias or applicability concerns** | **Discrimination (or other analysis of association with outcome performed)** | **Calibration*** | **Study conclusion** |
| --- | --- | --- | --- | --- | --- | --- | --- | --- |
| Ayami MS 2018 (All) ^37^ | P-PASS  <17, ≥17 | 327 pancreas recipients at a single centre, Gernany (2002-2015) | 1-, 5-,10-year pancreas, kidney and patient survival | Complete case analysis (Only 98.5% of cohort with PDRI and P-PASS) | No treatment details  No baseline demographics of predictor variables in cohort  No comparison of baseline data to derivation study  Different PDRI cutoffs (and not analysed as a continuous model)  Donor race set to Caucasian | NI | Observed: 72 pancreas failed  Predicted: NI | P-PASS not associated with all outcomes |
| Blok JJ 2016 (All) ^8^ | P-PASS  <17, ≥17 and as CN | 349 pancreas recipients at a single centre, Germany (1984-2013) | 1-, 5-, 10-year pancreas survival (DC) | Complete case analysis (Only 90.8% and 98.6% of cohort with P-PASS and PDRI respectively) | No treatment details  Donor race set to Caucasian as not collected by Eurotransplant,  No comparison of baseline data to derivation study  Different cutoffs for PDRI (median) | NI | Observed: 54 pancreas failed at 1 year  Predicted: NI | P-PASS no association with pancreas survival either with cut-offs or as CN model |
| Franz C 2019 (SPK, PAK) ^38^ | P-PASS  <17, ≥17 | 108 pancreas recipients at a single centre, Germany (2000-2017) | 1-, 5-year pancreas survival (DC) | NMD | No comparison of baseline data to derivation study  PDRI with different cut-offs from original (and not analysed as a continuous model) | NI | Observed: 27 pancreas failed at 1 year  Predicted: NI | P-PASS not associated with all outcomes |
| Finger EB 2013 (All) ^12^ | PDRI (similar risk quintiles to derivation) | 1115 pancreas recipients at a single centre, USA (1998-2011) | 3-month pancreas failure (DC) | NMD | No comparison of baseline data to derivation study | PDRI values for outcomes  C-statistic:  0.58 worst 20%  0.55 worst 10% | Observed: 114 pancreas failed at 3 months  Predicted: NI | Increased PDRI associated with 3-month pancreas failure |
| Foltys DB 2011 (SPK) ^43^ | P-PASS  <17, ≥17 | 55 pancreas recipients at a single centre, Germany (1999-2010) | 10-year pancreas survival | NMD | Graft failure undefined  No comparison of baseline data to derivation study  No baseline demographics of predictor variables in cohort | NI | Observed: 29 pancreas failed at 10 years  Predicted: NI | P-PASS not associated with 10-year pancreas survival |
| Garcia-Roca 2011  (unclear) ^44^ | P-PASS  <17, ≥17 | 249 pancreas recipients at a single centre, Spain (2000-2010) | 1-, 5-year pancreas survival and post-transplant complication rate | NI | Only in abstract form  Graft failure undefined  Unclear if graft survival was death-censored  No baseline demographics of predictor variables in cohort | NI | NI | P-PASS not associated with 1-, 5-year pancreas survival |
| Horvath S 2015  (All) ^39^ | P-PASS  <17, ≥17 | 119 pancreas recipients from single centre, UK (period not clear) | 1-year pancreas survival | Complete case analysis  (Only 97% of cohort with P-PASS | Only in abstract form  Graft failure undefined  PDRI with different cutoffs  Unclear if graft survival was censored  No baseline demographics of predictor variables in cohort | NI | NI | P-PASS not associated with 1-year pancreas survival |
| Kopp W 2016 (pancreas donors)  ^40^ | PDRI (CN) | 10444 pancreas donors from Eurotransplant registry (2004-2014) | Donor pancreas accepted, procured, transplanted | Multiple imputation of CIT (20 rounds) to enable PDRI calculation  Complete case analysis (Only 93.7% of cohort with P-PASS) | No comparison of baseline data to derivation study  CIT for PDRI set to 12 hours  CIT for PDRI for transplanted pancreas imputed for 28.6% of cohort  Donor race set to Caucasian as not collected routinely | C-statistic for PDRI in donors:  Reported vs non-reported (0.78, 95% CI 0.77-0.78)  Accepted vs non-accepted (0.79, 95% CI 0.78-0.8)  Procured vs non-procured (0.76, 95% CI 0.75-0.77)  Transplanted vs non-transplanted (0.84 95% CI 0.83-0.84) | Observed: 4354 donor pancreas declined  Predicted: NI | PDRI associated with reported, accepted, procured and transplanted pancreas donors |
| Schenker P 2010 (All) ^10^ | P-PASS  <17, ≥17 | 405 pancreas recipients at a single centre, Germany (1994-2009) | 1-, 5-, 10-year pancreas, kidney, patient survival | Complete case analysis  (Only 69% of cohort with P-PASS) | Graft failure undefined  No treatment details  No comparison of baseline data to derivation study | NI | Observed: 84 pancreas failure at 1 year  Predicted: NI | P-PASS not associated with all outcomes |
| Smigielska K 2018 (All) ^11^ | P-PASS  ≤14, ≤16,  ≤18, >18 | 408 pancreas recipients at multiple centres, Poland (1998-2015) | 1-year pancreas survival | Complete case analysis  (Only 73% of cohort with PDRI and P-PASS) | No treatment details  No comparison of baseline data to derivation study  P-PASS with different cutoffs | AUROC for P-PASS with outcome: 0.566 | Observed: 139 pancreas failure  Predicted: NI | P-PASS associated with 1-year pancreas survival |
| Vinkers MT 2008  (All) ^48^ | P-PASS  <17, ≥17 | 909 pancreas recipients from the Eurotransplant registry (2002-2005) | 1-year pancreas survival (DC) | Complete case analysis  (Only 76% of cohort with outcome data) | Graft failure undefined  No treatment details  Same population as per P-PASS derivation^1^, but applying P-PASS to a different outcome to check for an association | NI | Observed: 135 pancreas failure at 1 year  Predicted: NI | P-PASS<17 associated with better 1-year pancreas survival vs P-PASS ≥17 |
| Woeste G 2010 (All) ^47^ | P-PASS  <17, ≥17 | 49 pancreas recipients from a single centre, Germany (2004-2010) | 1-year pancreas survival | Complete case analysis  (Only 94% of cohort with P-PASS) | Graft failure undefined  No comparison of baseline data to derivation study | NI | Observed: 7 pancreas failure at 1 year  Predicted: NI | P-PASS not associated with 1-year pancreas survival + post-op complications |
| Ziaja J 2011 (SPK) ^46^ | P-PASS  <17, ≥17 | 46 pancreas recipients at a single centre, Poland (2004-2010) | Early post-transplant complications (1-month) | NMD | No comparison of baseline data to derivation study | NI | Observed: 5 pancreas failure, 5 patients died, 10 patients requiring return to theatre  Predicted: NI | P-PASS associated with 1-month post-transplant complications |

*All graft survival is uncensored for death unless otherwise stated*

**Observed and expected outcomes are for 1-year pancreas survival for PDRI and donor pancreas acceptance for P-PASS (as they are both the outcomes PDRI and P-PASS were derived against respectively) unless otherwise specified*

***Based on mean/median PDRI for entire cohort*

*A: abstract, CIT: cold ischaemia time, CN: continuous, DC: death-censored (all studies non-DC unless stated), DP: donor pancreas, F: full-text, MC: multicentre, NI: no information, NMD: no missing data, O/E: observed and predicted (expected) ratio, PDRI: pancreas donor risk index, PGS: pancreas graft survival, P-PASS: pre-procurement pancreas allocation suitability score, PTA: pancreas transplant alone, R: registry, SC: single centre, SE: standard error, UK: United Kingdom, USA: United States of America*
